# Hidden findings: How often does Holmium laser enucleation of the prostate (HoLEP) uncover prostate cancer?

**DOI:** 10.1007/s11255-025-04769-8

**Published:** 2025-08-31

**Authors:** Suraj Pursnani, Sri Saran Manivasagam, Ernesto Mohr, Abdul-Jawad Majeed, Mandy Hsu, Elizabeth Zook, Noor Banihashem Ahmad, Joaquín Gurovich, Rodrigo Cañas, Erik B. Lehman, Rodrigo Ledezma, Jay D. Raman

**Affiliations:** 1 Department of Urology, Penn State College of Medicine, 500 University Dr., Hershey, PA 17033 USA; 2 Department of Urology, Clinical Hospital of the University of Chile, Independencia, USA; 3 Department of Public Health Sciences, Penn State College of Medicine, 500 University Dr, Hershey, PA 17033 USA

**Keywords:** Incidental prostate cancer, HoLEP, Benign prostatic hyperplasia, Clinically significant prostate cancer, Racial factors

## Abstract

**Purpose:**

Benign prostatic hyperplasia (BPH) is a common condition with an increasing prevalence that parallels aging. Surgical procedures involving removal of the prostate can lead to detection of incidental prostate cancer (iPCa). Following holmium laser enucleation of the prostate (HoLEP), the incidence of such cases ranges from 5.6 to 23.3%. This study aims to provide a contemporary incidence of iPCa at HoLEP and associated risk factors.

**Methods:**

A multi-national retrospective cohort analysis of 579 patients who underwent HoLEP between 2021 and 2024 was performed. Prior to HoLEP, MRI was performed in 115 (20%) patients, of which 19 were diagnosed with PCa via conventional biopsy and were excluded from analysis. Clinical, demographic, and radiologic parameters were queried to determine association with iPCa and rates of clinically significant prostate cancer (csPCa).

**Results:**

Of 560 HoLEP patients, 39 (7.0%) were found to have de novo PCa, of which 13 (2.3%) had csPCa at the time of surgery. Patients with iPCa were more likely to be of white race (59% vs 29%, *p* < 0.001), ASA Grade 3/4 (41% vs 22%, *p* < 0.001), and higher preoperative mean PSA (5.7 vs 4 ng/dl, *p* = 0.007). On multivariate analysis, only white race (OR: 1.29, *p* = 0.04) and HoLEP morcellation time (OR: 0.91, *p* = 0.03) were associated with iPCa diagnosis.

**Conclusions:**

In an international HoLEP cohort, de novo PCa was detected in 7.0% of patients, with only 2.3% harboring clinically significant (≥ GG2) disease. Higher proportions of white men, men with ASA 3/4, and higher preoperative mean PSA were observed in iPCa. These data are valuable for appropriate preoperative patient counseling.

## Introduction

Benign prostatic hyperplasia (BPH) is a highly prevalent condition that increases steadily with age, affecting approximately 50% of men in their 60s and up to 80% of men in their 90s [1]. A range of medical and surgical treatments are available for BPH, with transurethral resection of the prostate (TURP) historically considered the gold standard [2]. Since its introduction in 1990, holmium laser enucleation of the prostate (HoLEP) has gained widespread adoption and is increasingly regarded as a new gold standard. HoLEP and thulium laser enucleation of prostate (ThuLEP) are the only surgical modalities endorsed by the AUA guidelines for use across all prostate sizes [3].

Surgical removal of prostate tissue—whether through TURP or HoLEP—can reveal incidental prostate cancer (iPCa). The incidence of iPCa after TURP has been reported to be approximately 4.16% [4]. For HoLEP, reported iPCa rates vary more widely, ranging from 5.64 to 23.3% across multiple studies [5–7].

Relatively few studies have examined predictive factors for iPCa following HoLEP. Bhojani et al. identified older age and elevated preoperative PSA as significant predictors [8]. Shevroy et al. found that larger prostate size was protective against iPCa [9]. In a review of 654 patients, Ohkawa et al. reported a 6.3% iPCa incidence and noted diabetes as a predictive factor [10]. In contrast, Porto et al. identified hypertension as an independent predictor [11]. Notably, no prior studies have investigated racial or ethnic variation in the incidence of iPCa following HoLEP.

Given the variation in findings across prior studies and the lack of data on racial differences, we aimed to provide a contemporary estimate of iPCa incidence and to identify associated risk factors in a diverse, multi-national HoLEP cohort. These findings may help inform future clinical decision-making and patient counseling.

## Methods

### Patient selection

We conducted a multi-institutional, multi-national retrospective cohort study of patients who underwent holmium laser enucleation of the prostate (HoLEP) between 2021 and 2024 at two centers—one in Chile with 364 patients and one in the United States with 196 patients. Institutional Review Board approval was obtained at both sites. All patients who underwent HoLEP during the study period were eligible for inclusion. Patients were excluded from analysis if they had a positive preoperative prostate biopsy. After applying exclusion criteria, data were collected on demographic characteristics, preoperative laboratory results, imaging findings, operative reports, pathology results, and postoperative outcomes. The covariates included in the study have been summarized in Table 1.

**Table 1:** Covariates and factors included in study

*Demographics*
Age (years)
Race
*Comorbidities*
BMI > 30 kg/m^2^
Diabetes mellitus
Ischemic heart disease
Chronic obstructive pulmonary disease
Stroke
Chronic kidney disease
American Society of Anesthesiologists Grade 3/4
*Laboratory, imaging and urodynamic parameters*
Maximum flow rate, Qmax (ml/s)
Postvoid residual urine (ml)
Prostate size (g)
Prostate-specific antigen (ng/dl)
Pre-operative MRI
*Operative parameters*
HoLEP enucleation time
HoLEP morcellation time

### Surgical technique

The procedure was performed under general anesthesia. After initial cystoscopy, 550 micron holmium laser fiber was used for enucleation with initial incision placed just proximal to the verumontanum. The plane between the capsule and the adenoma was then developed. After dissection of tissue on the right, the dissection was continued to the anterior apex and the left side. Following this, the prostatic adenoma was enucleated with en-bloc technique from the verumontanum circumferentially. The tissue was then removed using a morcellator. A 22 French 3-Way Foley catheter was placed and inflated with 50 cc of normal saline. Continuous bladder irrigation was continued for the next 24 h followed by catheter removal on postoperative day 2.

### Statistical analyses

Statistical analyses were performed using SPSS version 30. Continuous variables were expressed as means and standard deviations (SD) or as medians and interquartile ranges, depending on data distribution. Comparisons of continuous variables between groups were performed using analysis of variance (ANOVA) or independent samples *t*-tests, as appropriate. Categorical variables were compared using the Chi-square test. Logistic regression analyses were used to evaluate associations between clinical and demographic variables and the presence of incidental prostate cancer (iPCa). A *p*-value < 0.05 was considered statistically significant.

## Results

579 patients underwent HoLEP between 2021 and 2024. Prior to HoLEP, MRI was performed in 115 patients, with PI-RADS 4 and 5 lesions found in 21 (18.3%) patients. Prostate biopsy was performed in 105 patients and 19 (18.1%) patients had positive pathology for PCa, with 7 (6.7%) patients showing clinically significant cancer (grade group ≥ 2) on biopsy.

**Fig. 1:**
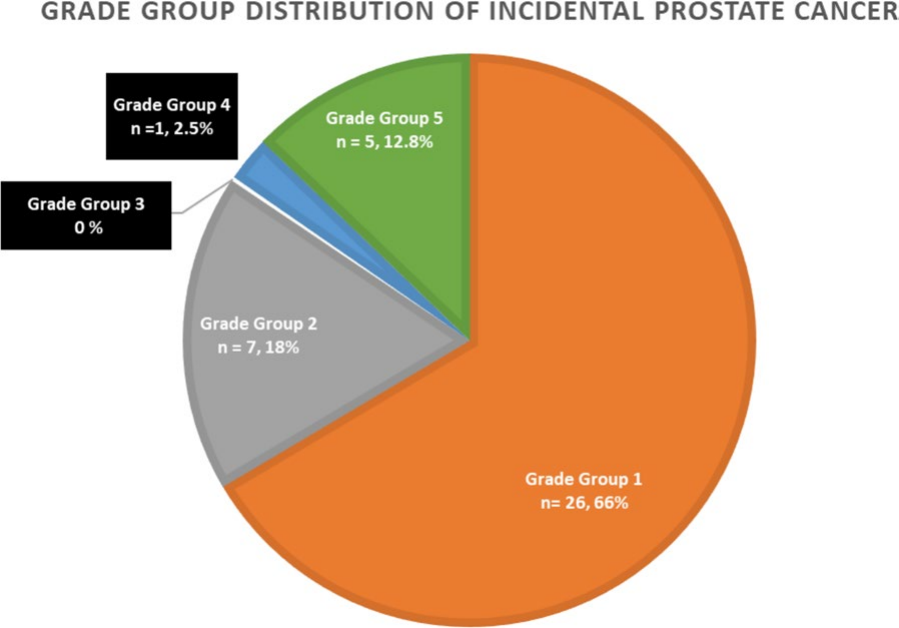
Grade group distribution of incidental prostate cancer

Of the 560 remaining patients without a PCa diagnosis, 39 (7.0%) were found to have de novo incidental prostate cancer (iPCa) on HoLEP specimens and 13 (2.3%) had clinically significant prostate cancer (csPCa) (≥ Grade Group 2). The specific histologic distribution of PCa is summarized in Fig. 1. Two-thirds of patients with iPCa had low-grade (GG1) disease. Notably, approximately 15% were diagnosed with GG4 or greater disease.

Among the 560 HoLEP patients, prostate magnetic resonance imaging (MRI) was conducted in 97 patients, while 85 patients underwent prostate biopsy prior to the procedure. The incidence of incidental prostate cancer (iPCa) among patients with a history of prostate MRI was 9.3% (9 of 97 cases), which did not differ significantly from the incidence observed in patients without prior MRI (6.3%, 29 of 463 cases; *p* = 0.29). Similarly, prior prostate biopsy did not significantly influence the detection rate of iPCa. The incidence among patients with a history of biopsy was 5.9%, compared to 6.9% in those without prior biopsy (*p* = 0.709). Of these 39 cases of iPCa, MRI was performed in 9 cases (23.1%). Among these, 2 patients had negative MRI (22.2%), 2 patients had PI-RADS 1–2 lesions (22.2%), and 5 patients had PI-RADS 3 lesions (55.5%).

Table 2 summarizes clinical, demographic, and operative characteristics of our study cohort with stratification between patients diagnosed with benign pathology and incidental prostate cancer at HoLEP. There was no clinically significant difference in age, BMI, and co-morbidities between groups of patients who were diagnosed with iPCa at HoLEP. However, the distribution of individuals belonging to white race (59% vs 28.8%, *p* < 0.001), having an ASA Grade 3/4 (41% vs 21.9%, *p* < 0.001), median preoperative PSA (5.7 vs 4, *p* = 0.007), and median IPSS score (4 vs 3, *p*-value = 0.074) was observed to be higher in those with iPCa. The distribution of patients who had undergone preoperative MRI (23% vs 16.8%, *p* = 0.29) and prostate biopsy (12.8% vs 15.3%, *p* = 0.709) was not different between those who were diagnosed with incidental prostate cancer. Interestingly, among operative characteristics, median HoLEP enucleation time (50 vs 55 min, *p* = 0.03) and HoLEP morcellation time (20 vs 25 min, *p* = 0.04) were significantly lower in those with iPCa.

**Table 2:** Comparison of clinical, demographic and operative characteristics between groups with benign pathology and incidental prostate cancer at HoLEP

	All cases (*n* = 560)	Benign pathology at HoLEP (*n* = 521)	Prostate cancer diagnosed at HoLEP (*n* = 39)	*p*-value
Age in years, median, IQR	68 (62–74)	68 (62–74)	72 (66–76)	0.34
Race (%)				
Black	8 (1.4%)	6 (1.2%)	2 (5.1%)	< 0.001
Hispanic	369 (65.9%)	354 (68.1%)	14 (35.9%)	
White	173 (30.9%)	150 (28.8%)	23 (59%)	
BMI > 30 kg/m^2^ (%)	343 (61.4%)	320 (61.4%)	22 (56.4%)	0.67
DM (%)	107 (19.1%)	97 (18.6%)	10 (25.6%)	0.52
Ischemic heart disease (%)	111 (19.8%)	133 (25.6%)	8 (20.5%)	0.35
COPD (%)	24 (4.3%)	22 (4.2%)	2 (5.1%)	0.79
Stroke (%)	25 (4.5%)	22 (4.2%)	3 (7.7%)	0.31
CKD (%)	39 (7.0%)	34 (6.5%)	5 (12.8%)	0.14
ASA 3/4 (%)	130 (23.3%)	114 (21.9%)	16 (41%)	< 0.001
IPSS score Median, IQR	3 (3–4)	3 (3–4)	4 (3–4)	0.07
*Q*_max_ (ml/s) Median, IQR	4 (2–4)	4 (2–4)	4 (1–4)	0.26
PVR, ml Median, IQR	9 (6–10)	9 (6–10)	8 (5–10)	0.11
Prostate size, g Median, IQR	97 (73–124)	97 (73–124)	84 (74–126)	0.71
PSA, ng/dl Median, IQR	4 (2.1–6.8)	4 (2.1–6.6)	5.7 (2.1–10.8)	0.007
Pre-operative prostate MRI	97, 17.3%	88, 16.8%	9, 23%	0.291
Pre-operative prostate biopsy	85, 15.1%	80, 15.3%	5, 12.8%	0.709
HoLEP enucleation time, min Median, IQR	55 (38–75)	55 (38–76)	50 (29–67.5)	0.03
HoLEP morcellation time, min Median, IQR	25 (15–35)	25 (15–37.2)	20 (10–27)	0.04

*BMI* Body Mass Index, *DM* diabetes mellitus, *COPD* chronic obstructive pulmonary disease, *CKD* chronic kidney disease, *ASA* American Society of Anesthesiologists, *IPSS* International Prostate Symptom Score, *MRI* magnetic resonance imaging, *Q*_*max*_ maximum Flow rate, *PVR* post-void residual urine, *PSA* Prostate-specific antigen

Univariate regression analysis found white race (OR 1.33, 95% CI 1.14–6.56, *p* = 0.04) and age (OR 1.09, 95% CI 1.02–1.16, *p* = 0.01) to be associated with increased odds of iPCa. Morcellation times were inversely associated with iPCa (OR 0.95, 95% CI 0.89–0.99, *p* = 0.04). On multivariate regression, neither age, BMI > 30 kg/m^2^, preoperative PSA, prostate size, prior MRI, prior prostate biopsy, or HoLEP enucleation time were associated with increased incidence of iPCa. However, white race (OR: 1.29, 95% CI = 1.14–9.32, *p*-value = 0.04) and HoLEP morcellation time (OR: 0.91, 95% CI = 0.78–0.94, *p*-value = 0.03) were significantly associated with incidence of iPCa (Table 3).

**Table 3:** Univariate and multivariate logistic regression analysis of the association between clinical and demographic variables and incidental prostate cancer at time of HoLEP

	Univariate analysis		Multivariate	
Parameter	Odds ratio (95% CI)	*p*-value	Odds ratio (95% CI)	*p*-value
Age (per 1 year)	1.09 (1.02–1.16)	0.01	1.06 (0.97–1.15)	0.15
Race:				
Hispanic	1		1	
White	1.33 (1.14–6.56)	0.04	1.29 (1.14–9.32)	0.04
Black	0.69 (0.24–1.09)	0.68	0.72 (0.18–5.68)	0.88
BMI > 30 kg/m^2^	0.38 (0.12–1.18)	0.09	0.55 (0.22–1.48)	0.62
ASA 3/4	2.94 (0.96–8.9)	0.06	3.08 (0.106–8.84)	0.38
Prostate size (per 1 g)	0.99 (0.97–1.00)	0.19	1.007 (0.99–1.02)	0.39
PSA	1.03 (0.99–1.07)	0.08	1.06 (0.99–1.14)	0.07
HoLEP enucleation time	0.98 (0.96–1.00)	0.16	0.99 (0.96–1.02)	0.79
HoLEP morcellation time	0.95 (0.89–0.999)	0.04	0.91 (0.78–0.94)	0.03
Prior MRI	1.52 (0.69–3.32)	0.29	1.77 (0.43–7.17)	0.42
Prior prostate biopsy	0.83 (0.31–2.19)	0.70	0.88 (0.15–5.14)	0.88

*BMI* Body Mass Index, *ASA* American Society of Anesthesiologists, *PSA* prostate-specific antigen, *MRI* magnetic resonance imaging, *PI-RADS* Prostate Imaging and Data Reporting System

## Discussion

Despite widespread prostate cancer screening, incidental prostate cancer (iPCa) remains a clinically relevant finding following surgery for benign prostatic hyperplasia (BPH) [12]. In our multi-national, multi-institutional study, the incidence of iPCa was 7.0%, with clinically significant prostate cancer (csPCa, ≥ Grade Group 2) observed in 2.3% of patients. This aligns with the broad range reported in the literature (5.6–23.3%), highlighting the continued variability in detection across studies [5–7].

While previous research has often emphasized age and PSA as predictors of iPCa, our study did not find age to be a significant factor. Bhojani et al. reported age and PSA as independent predictors [8], while Guo et al., in a meta-analysis of 23 studies, found neither age nor prostate volume to be associated with iPCa [13]. Our data corroborated the association with PSA but not age or prostate size. Importantly, our study is among the first to identify a significant association between white race and iPCa (OR 1.29, p = 0.04), contrasting with Porto et al., who found no racial association [11]. This suggests potential geographic or population-based differences that warrant further study.

Comorbidities such as diabetes and hypertension have been previously linked to iPCa, and our analyses found higher ASA grade to be more common in patients with iPCa. In a review of 654 patients, Ohkawa et al. reported a 6.3% iPCa incidence and noted diabetes as a predictive factor [10]. In contrast, Porto et al. identified hypertension as an independent predictor [11]. Notably, no prior studies have investigated racial or ethnic variation in the incidence of iPCa following HoLEP. However, these associations did not persist in multivariate analysis. Notably, higher preoperative PSA was significantly associated with both iPCa, reinforcing its utility as a predictive marker.

Operative characteristics also offered insight. We found that patients with iPCa had shorter HoLEP enucleation and morcellation times, though only morcellation time remained a significant predictor on multivariate analysis (OR 0.91, *p* = 0.03). These findings may reflect technical factors, gland characteristics, or disease biology that influence cancer detection.

Our results are consistent with prior evidence suggesting HoLEP has high sensitivity for detecting iPCa compared to other surgical modalities. In a matched-pair analysis, Rosenhammer et al. found HoLEP detected more iPCa cases than bipolar TURP, likely due to greater tissue removal [14]. Additional studies show HoLEP has comparable detection rates to open prostatectomy and may outperform Aquablation [15, 16]. Thus, HoLEP appears at least non-inferior to other tissue-excision techniques for iPCa detection.

The clinical implications of iPCa diagnosis after HoLEP remain under discussion. Most cases are low risk (Grade Group 1), as seen in 66.7% of our cohort, and are typically managed with active surveillance [12, 17–19]. Multiparametric MRI (mpMRI) can aid in risk stratification and decision-making regarding surveillance versus treatment [20].

Importantly, patients remain at risk for prostate cancer even after HoLEP. The postoperative decline in PSA complicates future screening. Studies by Lambert et al. and Elmansy et al. propose using postoperative PSA nadir or PSA velocity to guide long-term surveillance, but standardized guidelines are lacking [21, 22]. This remains an important area for future research.

Our study has several strengths. It represents one of the largest contemporary series examining iPCa following HoLEP and includes a diverse, multi-national patient population. We evaluated a broad range of demographic, clinical, and operative variables—including race, which has rarely been studied in this context. However, limitations include its retrospective design and potential heterogeneity in surgical technique and perioperative protocols across centers. Furthermore, family history of prostate cancer is an important factor that has not been recorded in our study and may be associated with incidental prostate cancer. In addition, the study is limited by underrepresentation of men of black race in the study population, a group that has been associated with increased risk of prostate cancer. Despite these limitations, our findings contribute to the understanding of iPCa and underscore the need for personalized approaches to postoperative risk assessment and cancer surveillance.

## Conclusions

In this international HoLEP cohort, incidental prostate cancer (iPCa) was detected in 7.0% of patients, with only 2.3% having clinically significant disease. iPCa was more common among white men and those with higher ASA classifications. These findings add to the growing body of literature on iPCa and highlight the potential predictive role of race and comorbidity burden. This information may aid in preoperative risk stratification and patient counseling.

## Data Availability

Datasets generated in this study are not available for dissemination due to data-share agreements.
